# Passive sampling of polycyclic aromatic hydrocarbons with low-density polyethylene: Equilibration limitations in aqueous suspensions

**DOI:** 10.1007/s10661-024-13182-0

**Published:** 2024-10-14

**Authors:** Jialin Liu, Binlong Liu, Shuya Xie, Peter Grathwohl

**Affiliations:** 1 Department of Geosciences, University of Tübingen, Schnarrenbergstraße 94-96, 72076 Tübingen, Germany; 2 China National Institute of Standardization, Beijing, 100191 China

**Keywords:** Partition coefficients, Aqueous boundary layer, Sorption isotherms, Desorption

## Abstract

Polyethylene (PE) and other polymers are widely and successfully used as passive samplers for organic pollutants in the environment. This study provides high-resolution experimental data from batch shaking tests on the uptake, reversibility, and linear equilibrium partitioning of polycyclic aromatic hydrocarbons (PAHs) using two different PE sheets of 30 µm and 80 µm thickness. Kinetics for phenanthrene are well described by a mechanistic first-order model with mass transfer limited by an aqueous boundary layer (with a mean thickness of 170 µm). Equilibration in laboratory batch systems during uptake and desorption is very rapid with characteristic times of 1–2 h but this depends on the boundary condition, e.g., the ratio of PE mass to water volume. Therefore, equilibration of PE in other setups, e.g., in soil slurries or sediment suspensions, may take orders of magnitude longer because the boundary condition for PE changes from finite to infinite bath conditions (soil or sediment particles may keep the concentration in water almost constant). Solid precipitates for high molecular weight PAHs explain partition coefficients below expected values because of kinetic limitations in such a system. Nevertheless, passive sampling can be employed safely if such limitations are considered; furthermore, partition coefficients can be estimated accurately by empirical relationships (e.g., within 0.1 log unit) based on molecular weight, octanol/water partition coefficients, or subcooled liquid solubilities.

## Introduction

Polycyclic aromatic hydrocarbons (PAHs) are a group of persistent organic compounds that have received widespread attention from researchers due to their carcinogenic, teratogenic, and mutagenic potential (Billet et al., 2008; Kozielska, 2018; Liu et al., 2019a; Shen et al., 2018) and their ubiquitous occurrence in environmental compartments such as atmosphere, water bodies, and soils (Qu et al., 2019). They typically occur in much higher concentrations than other persistent organic compounds such as polychlorinated biphenyls or dioxins. In monitoring of environmental quality, passive sampling techniques gained much attention recently because they pre-concentrate contaminants and allow easy extraction of target compounds with only small amounts of solvent (Adams et al., 2007; ter Laak et al., 2008; Seethapathy et al., 2008; Lohmann, 2012; Jonker et al., 2015). In aqueous phase, partitioning-driven passive samplers monitor the freely dissolved concentration which is relevant for pollutant uptake in organisms and thus they serve as a measure of toxicity or bioavailability of organic compounds. Compared to active samplers, passive samplers allow long-term monitoring, and they do not require electricity or other infrastructure (Donald and Anderson, 2017; Zhang et al., 2011) . In past studies, many materials have been used as absorbents for monitoring PAHs in environmental media, including low-density polyethylene films (LDPE) (Bartkow et al., 2004, 2006; Liu et al., 2022; Meire et al., 2019; Paulik et al., 2018), polyurethane foam (PUF) devices (Bohlin et al., 2010; Strandberg et al., 2018; Thang et al., 2020; Tuduri et al., 2006), and some common items in daily life, such as masks (Chan et al., 2021), silicone materials (Paulik et al., 2018; Sedlačková et al., 2021), and window films (Huo et al., 2019; Zhang et al., 2021). Among them, LDPE is the most readily available material and has been widely used in environmental media such as air, water, and soil (Booij et al., 2003; Adams et al., 2007; Fernandez et al., 2009; Lohmann, 2012; Mayer et al., 2014; Liu et al., 2019b; Meire et al., 2019; Meierdierks et al., 2022).

Besides applications in the field, passive samplers are also used in laboratory studies to study mass transfer of compounds from solids to water (desorption), equilibrium distribution, and biodegradation. This has the advantage of getting information of the freely dissolved concentration without the difficulties and artefacts commonly encountered in separation of water and solids (especially in soil and sediment slurry experiments). Often unclear or neglected is the influence of boundary conditions on uptake kinetics of target compounds or release of performance reference compounds from passive samples. Different boundary conditions exist in laboratory versus field but also between pure water and aqueous suspensions with a third phase (e.g., suspended sediments in rivers, wastewater, soil slurries, emulsions). The objectives of this study are follows: (i) to elucidate equilibration time scales for different boundary conditions (solid/liquid ratios, lab vs. field, particle suspensions, etc.) using coupled mass transfer models, (ii) to calculate the thickness of the aqueous boundary layer in uptake and desorption experiments; and finally (iii) to confirm sorption reversibility and linearity which is expected for a partitioning process. Large concentration ranges were employed to investigate the linearity of PE-water partition coefficients for different PAHs. Kinetic experiments were conducted with phenanthrene in high time resolution in both directions: sorption and desorption. Mechanistic mass transfer modelling was used to simulate passive sampling in soil or sediment slurries which takes much longer than in water alone. The study helps to understand mass transfer in passive sampling and to optimize monitoring of pollutants in different environmental systems. It may also explain the large differences observed in equilibration time scales for passive sampling in water (Seidensticker et al., 2017, 2019) which is much faster than in soil slurries (e.g., Meierdierks et al., 2022) because of the different boundary conditions as explained below.

## Materials and methods

### Chemical reagents

The organic solvents used for extraction, such as acetone (AC), ethyl acetate (EA), and cyclohexane (CH), were certified to GC grade purity. The standard solutions used to quantify the target compounds in the extracts were purchased from *Dr. Ehrenstorfer*; PAH MIX 31 and PAH MIX 14 were used as internal and external standards (PAH MIX 14 included non-deuterated EPA PAHs, while PAH MIX 31 included Nap-D_8_, Ace-D_10_, Phe-D_10_, Chr-D_12_, Py-D_12_, and EPA PAHs). Phenanthrene and phenanthrene-D_10_ standard samples were purchased from *Sigma-Aldrich Supelco* and *LGC Standards*, respectively. The PE ([C_2_H_4_]_n_) sheets were a commercial product and purchased from a hardware store (*Hornbach*). Abbreviations and physico-chemical properties of PAHs are given in Table 1.

**Table 1:** Physical–chemical properties of target PAHs used in this study

*Compound*	Abbr	MW	***S***_***sub***_^**a**^	log ***S***_***sub***_^**a**^	log ***K***_**ow**_^**b**^	log ***D***_**aq**_
		(g mol^−1^)	(mol m^−3^)	(kg L^−1^)	(-)	(m^2^ s^−1^)
*Naphthalene*	Nap	128	0.83	− 3.98	3.17	-
*Acenaphthylene*	Acy	152	0.29	− 4.35	3.94	-
*Acenaphthene*	Ace	154	0.13	− 4.71	4.15	-
*Fluorene*	Flu	166	0.075	− 4.91	4.18	-
*Phenanthrene*	Phe	178	0.024	− 5.38	4.35	− 9.12
*Anthracene*	Ant	178	0.028	− 5.30	4.35	− 9.11
*Fluoranthene*	Fla	202	0.0061	− 5.91	4.93	− 9.19
*Pyrene*	Py	202	0.0058	− 5.93	4.93	− 9.14
*Benz[a]anthracene*	BaA	228	0.0015	− 6.46	5.52	− 9.05
*Chrysene*	Chy	228	9.04E-04	− 6.69	5.52	− 9.21
*Benzo[b] & [k] fluoranthene*	BbF-BkF	252	3.29E-04	− 7.08	6.11	− 9.25
*Benzo[a]pyrene*	BaP	252	5.03E-04	− 6.90	6.11	− 9.05
*Indeno[1,2,3-cd]pyrene*	IP	276	1.81E-05*	− 8.30	6.7	− 9.28
*Dibenz[a,h]anthracene*	DBahA	278	1.44E-05*	− 8.40	6.99	− 9.25
*Benzo[ghi]perylene*	BghiP	278	1.80E-05*	− 8.30	6.7	− 9.28

^a^Subcooled water solubility (Eberhardt and Grathwohl, 2002)

^b^Octanol/water partition coefficient (Mackay et al., 1997)

^*^From EPISUITE

### Pretreatment of PE sheets

Two PE sheets of different thicknesses (30 µm and 80 µm) were subjected to a pre-cleaning step to remove potential background contaminants from the PE sheets before employment as passive samplers. PE sheets were immersed in 400 mL of cyclohexane in a 500-mL glass bottle, shaken on a horizontal shaker for at least 24 h, and transferred to another 500 mL glass bottle containing 400 mL of ethyl acetate, also shaken for at least 24 h. The PE sheets were then continuously rinsed with Millipore water to remove the solvent. The two PE sheets were cut into 1 × 1 cm pieces and stored immersed in Millipore water until use.

### Partition coefficients/sorption isotherms

Sorption isotherms were determined to investigate the linearity of uptake of PAHs onto PE sheets in a wide range of concentrations. Brown glass bottles (250 mL) with 0.4 g of PE sheets (pre-cleaned as described above) and 200 mL solution of different aqueous concentrations of PAHs containing 0.005 g of sodium azide (NaN_3_) were placed on a shaker at a frequency of 150 rpm for 7 days in the dark at a constant temperature of 20 °C. Initial concentration levels of PAHs were set to 120, 60, 30, 15, 7.5, 3, 1.5, and 0.75 µg L^−1^. Note that for the high molecular weight compounds, the highest concentration levels spiked would have exceeded the solid solubility limit (see Table 1) initially. Three parallel groups and one blank group were used for each concentration; blanks included PE only and ultrapure water with NaN_3_ to check for background contamination. For the determination of the PE-water partition coefficients (*K*_*PE*_), water and PE were extracted separately. An aliquot of water was sampled and liquid–liquid extracted by adding cyclohexane (for volumes, see Table 2) and 10 µL of the internal standard (PAH mix 31); mixtures were shaken for 30 min. Subsequently, the cyclohexane was transferred into clean vials. For PE extraction, the sheets were shaken with 10 mL of ethyl acetate twice for 24 h. Ten microliters internal standard was added to the combined extracts before adding an additional volume of 10 mL of cyclohexane. After shaking for 30 min, the cyclohexane was transferred into clean vials. In order to meet the detection limit of GC–MS, extraction methods were optimized by adjusting the sample and solvent volumes as shown in Table 2. Finally, target compounds were quantified with GC/MS. Recovery rates for low molecular weight PAHs (2–4 ring) were in the range of 90–102% (102% for Nap, 101% for Phe, 100% for Ant, 95% for pyrene) and for higher molecular weight PAHs (5–6 ring) 52–72% (62% for BbF-BkF, 53% for BaP, 52% for IP, 72% for BghiP). Independently measured concentrations in water (*C*_*w*_) and PE (*C*_*PE*_) were used to calculate *K*_*PE*_:

**Table 2:** Overview on extraction methods targeting different concentration levels

Low concentrations (< 10 µg L^−1^)	High concentrations (> 10 µg L^−1^)
For initial concentration:	
1. Sample 50 mL 2. Add 10 mL cyclohexane and 10 µL internal standards 3. Shake for 30 min and rest overnight 4. Transfer all cyclohexane to a glass sample vial and Barkey to 0.3 mL and transfer to autosampler vials	1. Sample 20 mL 2. Add 5mL cyclohexane and 10 µL internal standards 3.Shake for 30 min and rest overnight 4.Transfer 0.3 mL cyclohexane phase to autosampler vials
For equilibrium concentration:	
1. Take of 100 mL solution 2. Add 10 mL cyclohexane and 10 µL internal standards 3. Shake for 30 min and rest overnight 4. Transfer all cyclohexane to a glass vial and Barkey to 0.3 mL and transfer to autosampler vials	1. Take of 100 mL solution 2. Add 10 mL cyclohexane and 10 µL internal standards 3. Shake for 3 min and rest overnight 4. Transfer 0.3 mL cyclohexane phase to autosampler vials
Extraction of PE sheets:	
1. Sample ca. 0.2 g PE 2. Add 10 mL ethyl acetate, shake for 24 h 3. Repeat once 4. Add 10 µL internal standards 5. Transfer into 10 mL cyclohexane (over water) 6. Barkey to around 0.3 mL and transfer to autosampler vials	1. Sample ca. 0.1 g PE 2.Add 10 mL ethyl acetate, shake for 24 h 3. Repeat once 4. Add 10 µL internal standards 5. Transfer into 10 mL cyclohexane (over water) 6. Transfer 0.3 mL cyclohexane to autosampler vials

1 $${K}_{PE}={C}_{PE}/{C}_{w}$$

Concentration levels in water were chosen to allow the same activity of all PAHs under equilibrium conditions. Thus, fixed initial concentrations (*C*_*w,ini*_) were used for all compounds; since *K*_*PE*_ times, the water solubility (here *S*_*sub*_) is more or less constant (0.027 kg kg^−1^ according to Lohmann, 2012); theoretically, all compounds reach the same activity in water (*C*_*w*_/*S*_*sub*_):

2 $$\frac{{C}_{w}}{{S}_{sub}}=\frac{{C}_{w,ini}}{{S}_{sub} \left(1+{K}_{PE}\frac{{m}_{PE}}{{V}_{w}}\right)}\approx \frac{{C}_{w,ini}}{{S}_{sub} {K}_{PE}\frac{{m}_{PE}}{{V}_{w}}}$$

Therefore at a given *C*_*w,ini*_, *C*_*w*_/*S*_*sub*_ at equilibrium is constant for all PAHs. For the partitioning experiments, the maximum equilibrium *C*_*w*_/*S*_*sub*_ was 0.002 (0.044 in the kinetic experiments because of lower solid to liquid ratio) and thus below the solubility limits confirming an infinitely diluted solution. However, this experimental setup leads to high initial concentrations and the solubility limit may have been exceeded for high molecular weight PAHs causing precipitation of solids; such a third phase can slow down uptake of PAHs in PE as discussed below for a three-phase system (PE + solids + water).

### Uptake and desorption kinetics

Mass transfer kinetics was investigated with 80-µm-thick PE sheets in batch experiment using phenanthrene (Phe) as representative compound of the PAHs. Phe shows relatively high concentrations among PAHs in the environment; it has an intermediate solubility and partition coefficient which makes it a representative PAH with an intermediate equilibration time.

For preparation of the Phe stock solution, solid Phe was dissolved in methanol to obtain a concentration of 1 g L^−1^. 1.2 mL of the stock solution was added to 2 L of ultrapure water resulting in an initial concentration of 600 µg L^−1^ (the solubility of solid Phe in water is 1150 µg L^−1^) and a final equilibrium concentration of around 50 µg L^−1^. 0.05 g L^−1^ NaN_3_ was added to prevent biodegradation. The aqueous solution was placed on a shaker for at least 5 h to ensure homogeneous mixing.

Clean 80-µm PE sheets of 0.25 g each and 500 mL of 600 µg L^−1^ Phe stock solution were added to a 500-mL brown glass bottle. The bottle was placed on a magnetic stirrer at 150 rpm in the dark and at a constant temperature of 20 °C. An aliquot of 1 mL of the aqueous solution was sampled initially and after 2, 4, 6, 8, 10, 15, 20, 30, and 40 min and after 1, 2, 4, 8, 24, and 48 h. The experiment was performed in triplicate; a control group with the same volume of Phe stock solution but no PE sheets allowed to monitor potential losses of Phe during the kinetic experiments. Average concentrations in the control group were 616 µg L^−1^ (standard deviation ± 25.5 µg L^−1^, *n* = 16) indicating no losses and high precision of measurements (coefficient of variation = 4%).

For analysis, 10 µL of the internal standard solution and 500 µL of cyclohexane were added to the 1 mL water samples; glass vials then were tightly capped with a polytetrafluoroethylene (PTFE)-coated silicone cap and shaken on a horizontal shaker for at least 40 min. Then, the vials were allowed to stand overnight and the upper portion of cyclohexane was transferred to a 0.3-mL GC autosampler vial. Finally, the PAH concentrations in the samples were quantified by GC–MS.

In order to check for potential sorption/desorption hysteresis, the experimental setup was finally reversed: 0.2 g of the PE sheets that had reached equilibrium in the uptake experiment was added to glass bottles containing 500 mL ultrapure water again in triplicate. The bottles were placed on a magnetic stirrer in the dark at a constant temperature of 20 °C. Sampling time points were 3, 5, 10, 17, 30, 45, and 90 min, and 3, 8, 13, 44, and 88 h. For sorptive uptake, the initial concentration of a control bottle sampled at each time step was used for the mass balance (concentrations were in average 617 µg L^−1^ (± 26 µg L^−1^, *n* = 16) and thus very close to the nominal value of 600 µg L^−1^). For desorption, a blank group containing the same volume of ultrapure water, but no PE sheets, were employed to determine potential cross contamination with Phe, which was less than 5% of the final equilibrium concentration, but higher at early low concentrations and thus was considered in the mass balance calculation (background correction).

### GC–MS analysis

Sixteen PAHs were used as target compounds for concentration determination by gas chromatography and coupled mass spectrometry (HP 6890 and HP 5973). The chromatographic column was 25 m × 0.25 mm × 0.25 µm coated with a 0.25-µm-thick film of 5% phenyl and 95% dimethylsiloxane (Agilent Technologies). The ramp-up procedure of the GC started at 65 °C and was maintained for 4 min, then the temperature was increased at a rate of 10 °C per min to 270 °C for 10 min, and finally a second constant temperature of 310 °C was held for 6 min. Helium was used as the inert carrier gas at a flow rate of 0.7 mL min^−1^ and the mass spectrometer was operated in selected ion mode. Both external standard (PAH mix 14) and internal standard (PAH mix 31) methods were used for the quantification of the target compounds PAHs.

## Experimental results

### Partition coefficients/sorption linearity

**Fig. 1:**
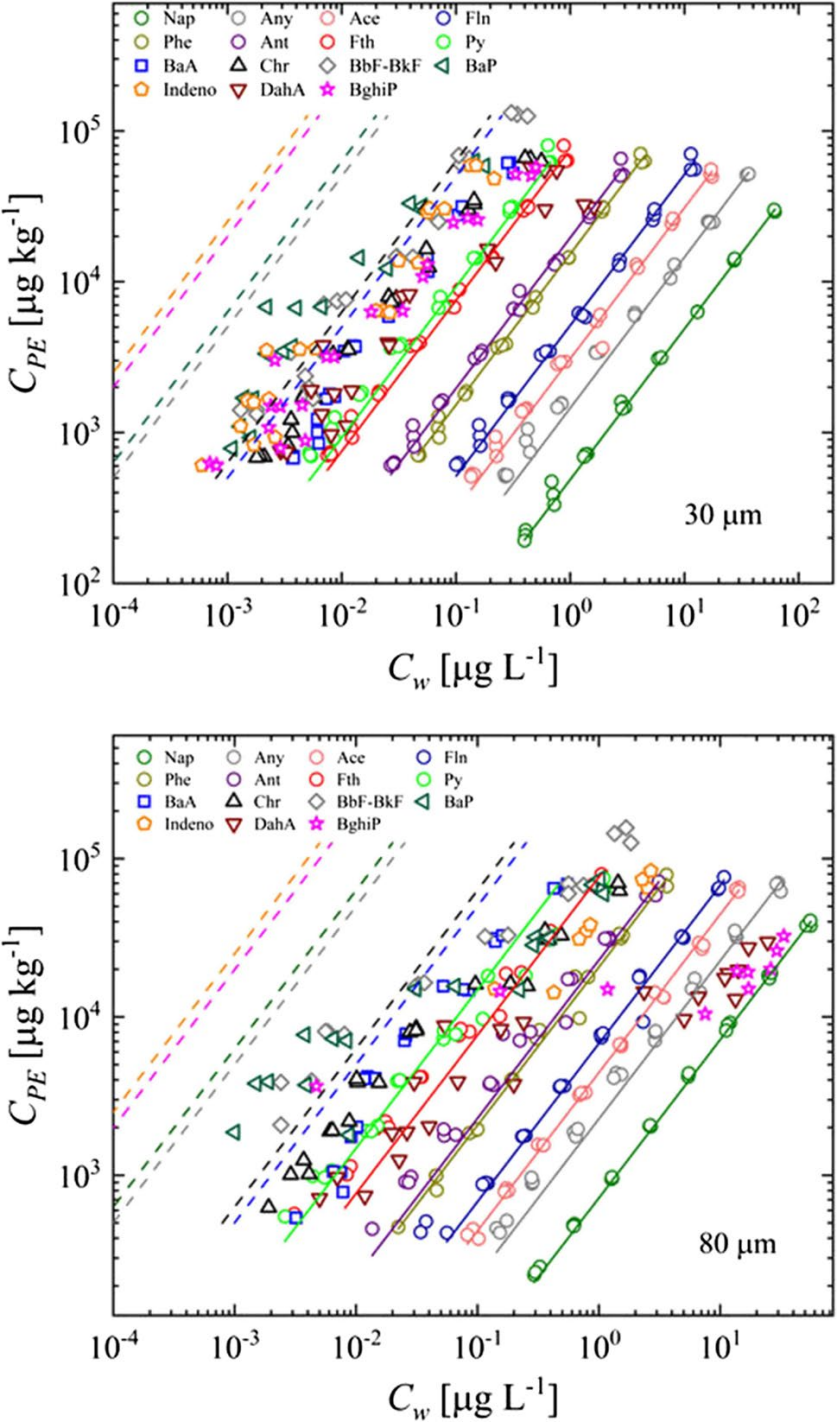
Partitioning experiment for uptake of PAHs on PE sheets of different thicknesses; solid lines represent average *K*_*PE*_ values from Nap (right) to Py (left), and dashed lines indicate expected uptake under equilibrium which was not reached for HMW PAHs

In partitioning, the interaction of a solute between a sorbent and water should be largely independent of the loading level over extended concentration ranges. Figure 1 shows concentrations measured separately in PE and aqueous phase for the 80 µm and 30 µm sheets (sorption isotherms). Uptake of PAHs increases significantly with the increase of molecular weight (*MW*) about two orders of magnitude if MW increases by 50%, with the weakest sorption for Nap and the strongest sorption for BaA (data of PAHs which has higher MW than BaA could not be evaluated because the equilibrium was not achieved potentially due to solid precipitates).

Sorption isotherms for Nap and BaA were perfectly linear confirming a partitioning mechanism over an equilibrium concentration range of 3 orders of magnitude. Table 3 lists *K*_*PE*_ calculated as ratio of measured *C*_*PE*_ and *C*_*w*_. For higher molecular weight (HMW), PAHs data scatter increases and *K*_*PE*_ values calculated become lower than expected especially at high concentration levels. Compounds with high *MW* or log *K*_*PE*_ > 6 (Indeno-BghiP) could not be evaluated because concentrations were below detection limits in water or equilibrium was not reached. For Chr, BbF-BkF and BaP as well as Py and BaA, the highest 3 and 2 concentration levels were excluded due to concerns about equilibration (and large data scatter). As discussed above, the experimental setup had to start at high concentrations in order to stay above the detection limit in water after equilibration because of strong sorption. This initially may have caused precipitation of HMW PAHs which would slow down uptake in PE as discussed later (then, uptake in PE is slower because the boundary conditions change to the infinite bath as long as the precipitated crystals dissolve). This difficulty might also be a reason why *K*_*PE*_ for HMW PAHs in literature mostly come from empirical relationships (Bao et al., 2012; Villar et al., 2018; Vrana et al., 2019) or are obtained in methanol/water mixtures (Smedes et al., 2009).

**Table 3:** Measured log *K*_*PE*_ values compared with literature data

	log *K*_*PE*_					
	This study		Lohmann (2012)	Cornelissen et al. (2008)	Fernandez et al. (2009)	Smedes et al. (2009)
Thickness	30 µm	80 µm		100 µm	25 µm	70 µm
Nap	2.71 ± 0.04	2.88 ± 0.02	2.9	3.04		2.8
Any	3.24 ± 0.07	3.44 ± 0.06	3.5			3.2
Ace	3.52 ± 0.07	3.65 ± 0.04	3.6			3.6
Fln	3.73 ± 0.05	3.87 ± 0.10	3.9	3.78		3.8
Phe	4.18 ± 0.03	4.33 ± 0.06	4.2	4.14	4.3	4.2
Ant	4.31 ± 0.05	4.46 ± 0.10	4.2	4.37	4.3	4.3
Fth	4.91 ± 0.05	5.01 ± 0.11	4.9	4.85	4.9	4.9
Py^a^	5.07 ± 0.07	5.17 ± 0.13	4.9	5.02	4.7	5.1
BaA^a^	5.34 ± 0.10	5.34 ± 0.16	5.6	5.62	5.5	5.7
Chr^b^	5.50 ± 0.07	5.48 ± 0.07	5.6	5.56	5.5	5.8
BbF-BkF^b^	5.78 ± 0.17	5.94 ± 0.21	6.2	6.06	6.3	6.7
BaP^b^	6.05 ± 0.19	6.07 ± 0.33	6.2	6.22	6.4	6.8
Indeno^c^						7.4
DahA^c^						7.3
BghiP^c^						7.3

^a^Two highest concentration levels excluded

^b^Three highest concentration levels excluded

^c^Equilibrium not reached

Table 3 lists the average equilibrium *K*_*PE*_ values incl. standard deviations calculated from all data points obtained (ratios of measured equilibrium concentrations in water and PE). Only data above the aqueous detection limits (0.001 µg L^−1^) and for HMW PAHs only low concentration levels were considered (because of risk of precipitation at high concentrations leading to non-equilibrium). *K*_*PE*_ values agree well with literature and increase with molecular weight of PAHs and decreasing solubility as expected. Results for Phe agree well with log *K*_*PE*_ obtained from the sorption–desorption kinetics (4.39) experiment. Log *K*_*PE*_ values for 30 µm and 80 µm PE sheets are very similar and indicate no accumulation of PAHs on the surface potentially leading to higher *K*_*PE*_ for the thinner 30-µm sheets as already reported by Lohmann (2012). In fact, *K*_*PE*_ for the 80-µm PE seems slightly higher. Lei et al. (2020) found that for LDPE sheets of the same thickness from two different companies, *K*_*PE*_ values may vary for many PAHs significantly. In contrast, Hale et al. (2010) reported *K*_*PE*_ values for Phe, Ant, and Py which are not significantly different for 51-µm and 26-µm-thick PE sheets.

**Fig. 2:**
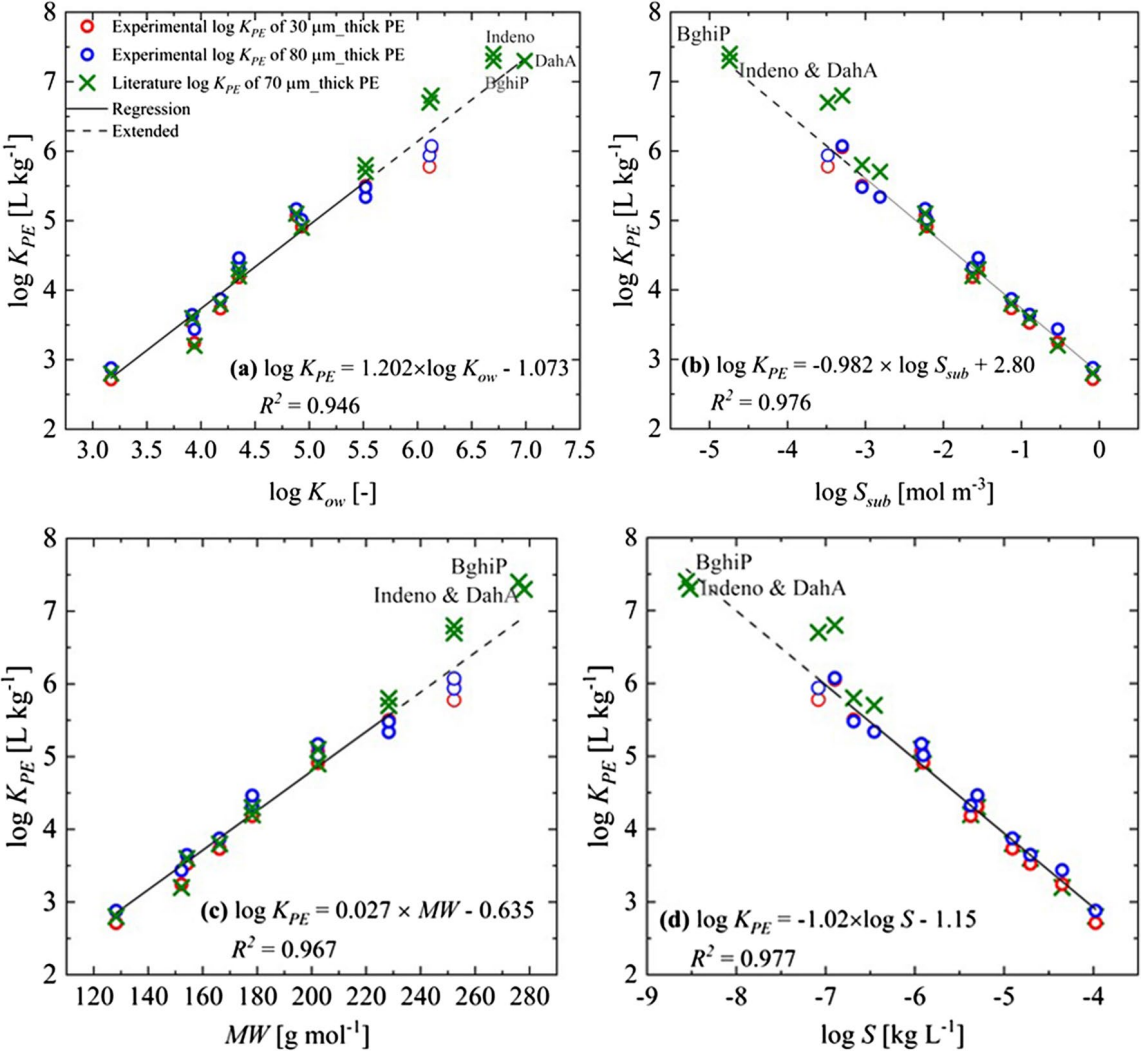
Relationships between log *K*_*PE*_ and log *K*_*ow*_, *MW*, log *S*_*sub*_ [mol m^−3^], and log *S* [kg L^−1^] (for standard deviations see Table 4); compounds with standard deviation > 0.15 log units excluded from regressions (BaP, BbF-BkF); green × symbols show literature data from Smedes et al. (2009), not included in regressions

Figure 2 shows relationships between measured log *K*_*PE*_ and log *K*_*ow*_, log *S*_*sub*_ as well as *MW* for Nap and BaP. Log *K*_*PE*_ increases almost linear with log *K*_*ow*_ and *MW* and correlate inversely with the subcooled liquid solubility (log *S*_*sub*_) with a slope close to − 1 as expected from partitioning theory (Raoult’s law) and reported by others (e.g., Zhu et al., 2015; Lohmann, 2012). Because of this inverse linear relationship, *K*_*PE*_ × *S*_*sub*_ is supposed to be constant; for Phe (as representative PAH), we get 0.074 kg kg^−1^; for comparison, Razzaque and Grathwohl (2008) reported 0.043 kg kg^−1^ for *K*_*oc*_ × *S* for organic carbon in soils and sediments. Table 4 lists empirical relationships to calculate log *K*_*PE*_ from this study and the literature and compares log *K*_*PE*_ calculations for Phe. These simple relationships work reasonably well for PAHs; for other compounds, poly-parameter linear relationships could allow better predictions (e.g., Khawar & Nabi, 2021).

**Table 4:** Empirical relationships of log *K*_*PE*_ vs. log *K*_*ow*_, log *S*_*sub*_, as well as *MW* for PAHs

Reference	Correlation	*n*^a^	*R*^2^	log* K*_*PE*_ (Phe)
Figure 2, *K*_*ow*_, this work	log *K*_PE_ = 1.20 (± 0.068) log *K*_ow_ -1.07 (± 0.307)	20	0.95	4.15
Lohmann (2012)	log *K*_PE_ = 1.22 (± 0.046) log *K*_*ow*_ - 1.22 (± 0.24)	65	0.84	4.09
Booij et al. (2003) (13 °C)	log *K*_PE_ = 0.97 log *K*_*ow*_ - 0.13	32	0.95	4.09
Adams et al. (2007)	log *K*_PE_ = 1.2 log *K*_*ow*_ - 0.97	8	0.95	4.25
Figure 2, *S*_*sub*_ [mol m^−3^], this work	log *K*_PE_ = − 0.93 (± 0.033) log *S*_sub_ + 2.80 (± 0.06353)	20	0.98	4.23
Lohmann (2012), *S*_*sub*_ [mmol L^−1^]	log *K*_PE_ = − 0.85 (± 0.023) log *S*_sub_ + 2.85 (± 0.071)	65	0.94	4.23
Figure 2, *MW*, this work	log *K*_PE_ = 0.027 (± 0.001) MW - 0.64 (± 0.217)	20	0.97	4.21
Lohmann (2012); Ma et al. (2010)	log *K*_PE_ = 0.025 (± 0.001) MW - 0.15 (± 0.217)	65	0.90	4.30
Figure 2, *S* [kg L^−1^], this work	log *K*_PE_ = − 1.02 (± 0.038) log *S* − 1.15 (± 0.326)	20	0.98	4.10
Lohmann (2012), *S* [kg L^−1^]	log *K*_PE_ = − 0.814 (± 0.038) log *S* - 0.56 (± 0.206)	65	0.88	3.70
Zhu et al. (2015), *S* [kg kg^−1^]	log *K*_PE_ = − 1.01 log *S* 0.95	63	0.88	4.33

^a^Number of observations

### Sorption/desorption kinetics

**Fig. 3:**
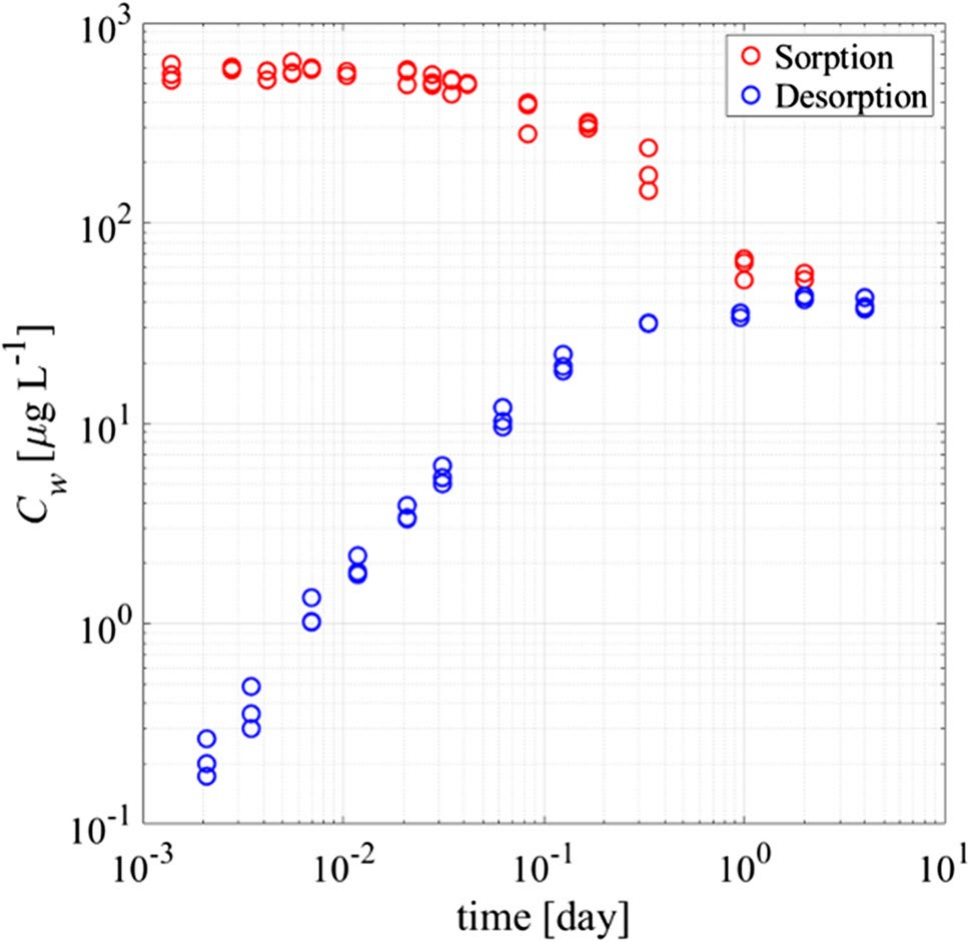
Concentrations vs. time for sorption/desorption of Phe from 80-µm PE sheets (*C*_*desorption*_ corrected for background values; only relevant for low concentrations; sorption, original measured concentrations)

As Fig. 3 shows, equilibrium for Phe was reached in less than 24 h in both experiments. In the uptake mode, Phe in the solution decreased from initial concentration of 630 µg L^−1^ (SD =  ± 7.4 µg L^−1^, *n* = 3) to 53.5 µg L^−1^, and ultimately, 91.5% of the initial Phe mass was taken up by the PE. During desorption, the concentration of Phe in the solution increased to 42 µg L^−1^ (with SD =  ± 2.1 µg L^−1^, *n* = 3) and 92% stayed in the PE. For desorption, initially a linear increase of the Phe concentration in aqueous phase is observed, whereas during sorptive uptake, aqueous concentrations decrease exponentially until the equilibrium concentration is reached, which perfectly corresponds to mass transfer limitations by diffusion through an aqueous boundary layer described by a first-order model (see Eq. 3).

### Fitting of mass transfer kinetics: *K*_*PE*_ and the aqueous boundary layer

Polyethylene is a polymer, which is easily penetrated by organic chemicals (Lei et al., 2020). Thus, mass transfer of strongly hydrophobic compounds is known to be limited by diffusion across an external boundary layer (Meierdierks et al., 2022; Seidensticker et al., 2017; ter Laak et al., 2008). The concentration of the target compound in the PE sheets changes with time depending on the passive sampler thickness (${d}_{PE}$), the partition coefficient (*K*_*PE*_), and the characteristic mass transfer coefficient (= *D*_*aq*_/*δ*_*aq*_; *D*_*aq*_ and *δ*_*aq*_ denote the aqueous diffusion coefficient and the thickness of the aqueous boundary layer, respectively). The relative solute mass which has diffused into or out of the passive sampler (*M*/*M*_*eq*_) then is:

3 $$\frac{M}{{M}_{eq}}=1-\text{exp}\left(-\frac{{D}_{aq}}{{\delta }_{aq}} \frac{2}{{K}_{PE} {\rho }_{PE} {d}_{PE}}\left(1+{K}_{PE}\frac{{m}_{PE}}{{V}_{w}}\right)t\right)$$

*M*_*eq*_ is the solute mass which has diffused into or out of the PE after equilibrium was reached. *ρ*_*PE*_ denotes the mean PE density (0.92 kg L^−1^); *m*_*PE*_ and *V*_*w*_ represent the mass of PE and the volume of water in the batch system. The benefit of the *M*/*M*_*eq*_ notation is its independence from mass transfer direction (sorptive uptake or desorption). In sorptive uptake, *M*/*M*_*eq*_ represents the relative concentration in the solid, while for desorption kinetics, it reflects the relative concentration in the aqueous phase. The term $\left(1+{K}_{PE}\frac{{m}_{PE}}{{V}_{w}}\right)$ only applies in finite bath conditions (e.g., batch experiments) and shows how equilibration gets faster with increasing solid to liquid ratio (*m*_*PE*_/*V*_*w*_). Under field conditions, particularly monitoring rivers, lakes, and oceans, *V*_*w*_ is often considered practically infinite (infinite bath conditions), which streamlines the equation ($\left(1+{K}_{PE}\frac{{m}_{PE}}{{V}_{w}}\right)$ drops out). Note that dissolved organic substances such as humic acids, proteins, or surfactants may lead to a decrease of the partition coefficient and thus may accelerate kinetics (te Laak et al., 2009) and in extreme cases may result in a shift of the mass transfer resistance into the polymer (Seidensticker et al., 2017).

Fitting of kinetics was based on *K*_*PE,a*_ (= *C*_*PE*_/*C*_*w*_), which at early times is less dependent on the solid to liquid ratio compared to *M*/*M*_*eq*_. Before equilibrium is established, an apparent partition coefficient (*K*_*PE,a*_) is observed as described by Eqs. 4 and 5 for sorptive uptake and desorption as a function of time *t*, respectively:

4 $${K}_{PE,a}=\frac{1-\text{exp}\left(-\frac{{D}_{aq}}{{\delta }_{aq}} \frac{2}{{K}_{PE} {\rho }_{PE} {d}_{PE}}\left(1+{K}_{PE}\frac{{m}_{PE}}{{V}_{w}}\right)t\right)}{1+{K}_{PE}\frac{{m}_{PE}}{{V}_{w}}\text{exp}\left(-\frac{{D}_{aq}}{{\delta }_{aq}} \frac{2}{{K}_{PE} {\rho }_{PE} {d}_{PE}}\left(1+{K}_{PE}\frac{{m}_{PE}}{{V}_{w}}\right)t\right)}{K}_{PE}$$

5 $${K}_{PE,a}=\frac{1+\frac{{V}_{w}}{{K}_{PE}{m}_{PE}}\text{exp}\left(-\frac{{D}_{aq}}{{\delta }_{aq}} \frac{2}{{K}_{PE} {\rho }_{PE} {d}_{PE}}\left(1+{K}_{PE}\frac{{m}_{PE}}{{V}_{w}}\right)t\right)}{1-\text{exp}\left(-\frac{{D}_{aq}}{{\delta }_{aq}} \frac{2}{{K}_{PE} {\rho }_{PE} {d}_{PE}}\left(1+{K}_{PE}\frac{{m}_{PE}}{{V}_{w}}\right)t\right)}{K}_{PE}$$

The exponential terms in Eqs. 3–5 approach 0 as time becomes sufficiently large (equilibrium: *K*_*PE,a*_ = *K*_*PE*_). If *K*_*PE,a*_ is known, *K*_*PE*_ and *δ*_*aq*_ for Phe can be fitted from the experimental data for sorptive uptake and desorption simultaneously.

**Fig. 4:**
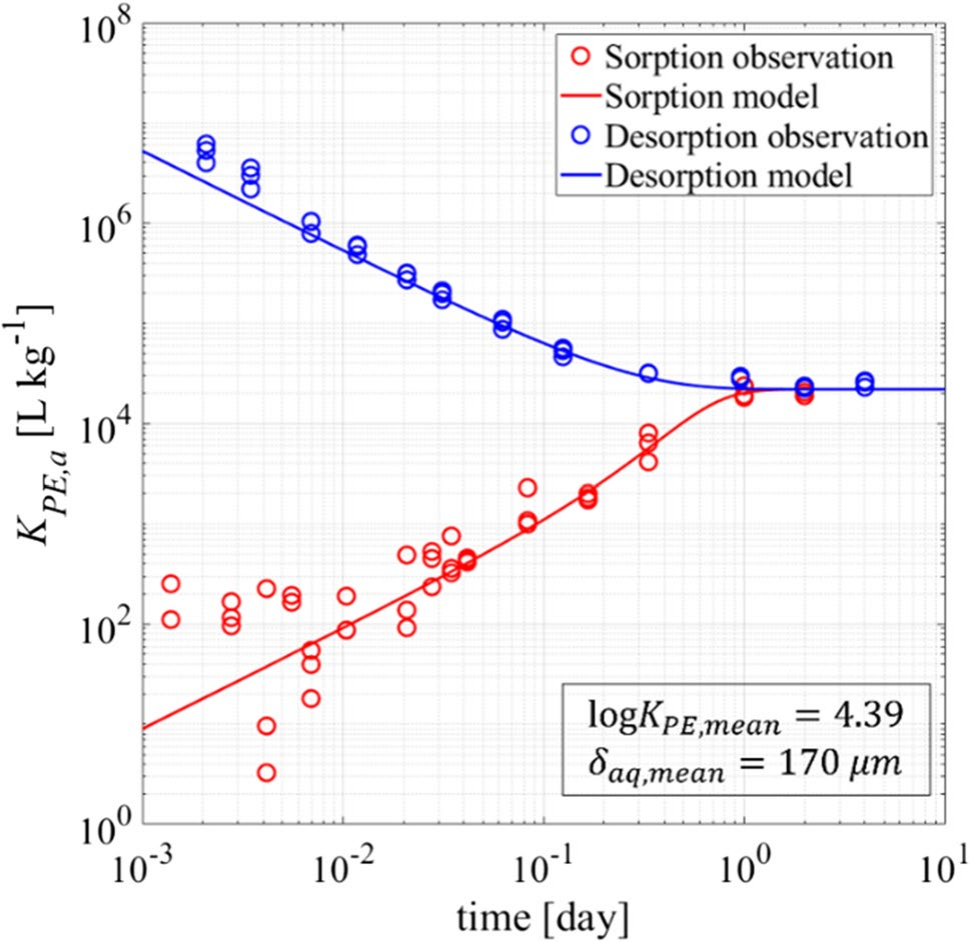
Apparent partition coefficients *K*_*PE,a*_ for sorptive uptake (red) and desorption (blue) versus time with different values of the solid to liquid ratio ${m}_{PE}/{V}_{w}$ (5E-5 kg L^−1^ for sorption and 4.65E-4 kg L^−1^ for desorption; other parameters: ${D}_{aq}=$ 6.71E-10 m^2^ s^−1^; ${\rho }_{PE}=$ 0.92 kg L.^−1^; ${d}_{PE}=$ 80 $\mu$ m)

As expected, *K*_*PE,a*_ for sorptive uptake increased with time, while it decreased for desorption, as shown in Fig. 4. After 24 h, experimental *K*_*PE,a*_ values stabilized indicating equilibrium conditions at log *K*_*PE*_ of 4.39. Thus, the results indicate that uptake and desorption of a specific target compound in PE are perfectly reversible. Additionally, Fig. 4 illustrates how error propagation in mass balances calculated (here only the aqueous phase is monitored) results in significant uncertainties in triplicates of low *K*_*PE,a*_ during sorptive uptake, as opposed to the situation with high initial *K*_*PE,a*_ during desorption and near equilibrium. This data scatter is not observed for aqueous concentrations depicted in Fig. 3. By jointly fitting Eqs. 4 and 5, *δ*_*aq*_ was determined to be 170 µm, while 163 µm and 193 µm were obtained for sorption and desorption when fitted separately (indicating an error of around 10%). Because of large uncertainty, early data (< 10 min) were not taken into account in fitting.

## Implications: Characteristic times for passive sampler equilibration in water and aqueous suspensions

The data presented in Figs. 3 and 4 demonstrate the applicability of the mechanistic first-order mass transfer model (Eq. 2) for simulating the uptake kinetics of various target compounds and the release of corresponding performance reference compounds. Furthermore, it allows the calculation of equilibrium time scales, represented as characteristic time (*t*_*ch*_) for various target compounds and PE to water ratios (*m*_*PE*_/*V*_*w*_). The characteristic time is the inverse of the rate constant used in the exponential term in Eq. 3.

6 $${t}_{ch}=\frac{{\delta }_{aq}}{{D}_{aq}} \frac{{K}_{PE} {\rho }_{PE} {d}_{PE}}{2 \left(1+{K}_{PE}\frac{{m}_{PE}}{{V}_{w}}\right)}$$

**Fig. 5:**
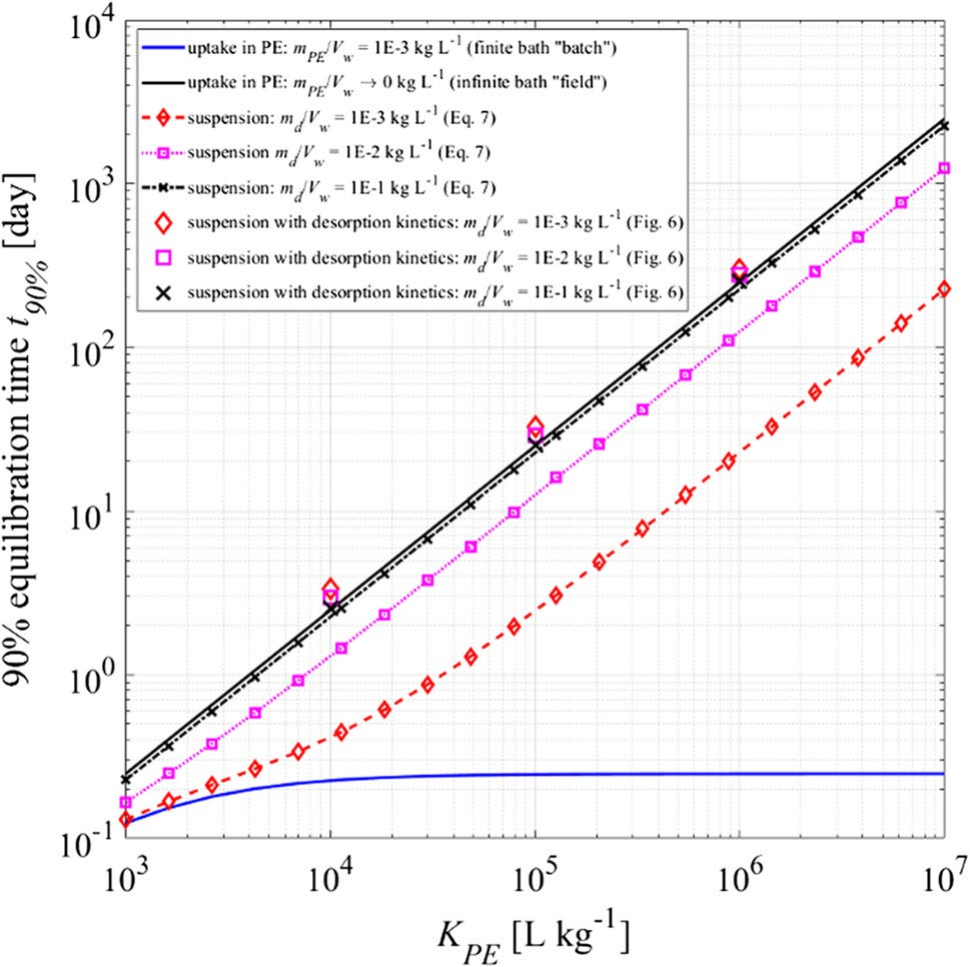
Time to achieve 90% of equilibrium (2.3 × *t*_*ch*_) between PE and water vs. *K*_*PE*_ for sorptive uptake batch experiments (finite bath) and field (infinite bath) as well as for passive sampling in suspensions (three phases: suspended solids + PE + water) for three solid to liquid ratios (*m*_*d*_/*V*_*w*_) according to Eq. 7 (film diffusion only); symbols only: kinetics simulated by a coupled film intraparticle pore diffusion model for particles of 100 µm size (see Fig. 6 for kinetic modelling parameters); *K*_*d*_, values ten times smaller than *K*_*PE*_

*t*_*ch*_ increases linearly with increasing PE (*d*_*PE*_) and film thickness (*δ*_*aq*_). In finite bath conditions, *t*_*ch*_ becomes independent on *K*_*PE*_ if the term *K*_*PE*_* m*_*PE*_/*V*_*w*_ (= mass fraction in PE compared to water under equilibrium) is much larger than 1 (but still gets smaller with increasing *m*_*PE*_/*V*_*w*_). In contrast, with a constant concentration in water (= infinite bath conditions) as, for example, used by Lei et al. (2020), equilibration time may be much longer. This also demonstrates that time scales in batch experiments may be much shorter than in the field (infinite bath, *V*_*w*_ → ∞). Figure 5 shows time scales for 90% equilibration (which takes 2.3 times longer as *t*_*ch*_) vs. *K*_*PE*_ for our experimental batch system and the infinite bath. In the laboratory, most compounds reach equilibrium within a day, but those with high *K*_*PE*_ values may require several years for full equilibration under field conditions (infinite bath). *D*_*aq*_ and *ρ*_*PE*_ are relatively constant for different PAHs or PE sheets and *K*_*PE*_ is decisive for *t*_*ch*_ for infinite bath conditions.

**Fig. 6:**
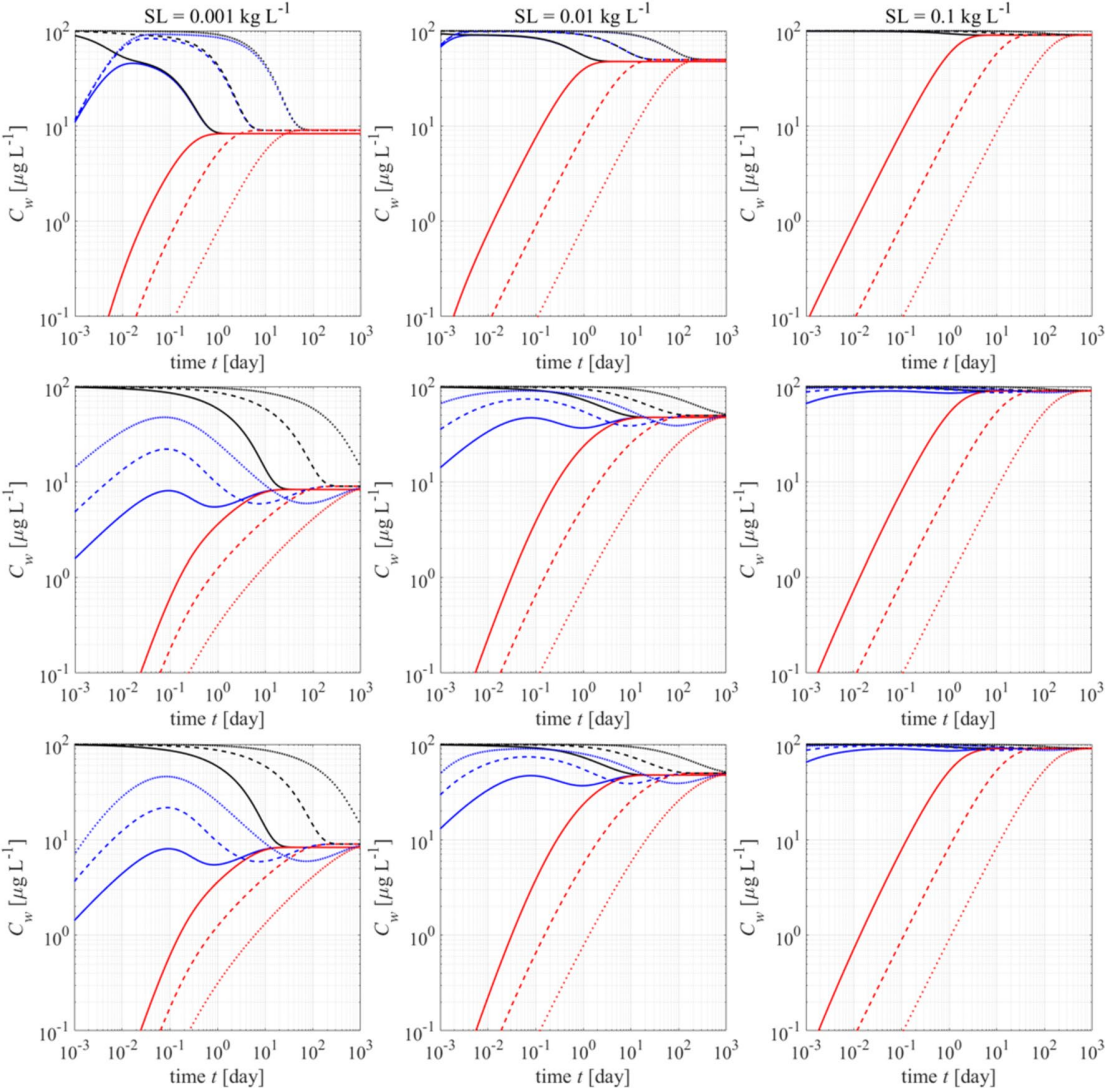
Redistribution dynamics of pollutants in water (blue) and PE (red) during desorption from solids (black declining lines) for 3 solid to liquid ratios (SL) and increasing values of the solid-water distribution coefficient *K*_*d*_ (1000: solid, 10,000: dashed, 100,000: dotted lines) and *K*_*PE*_ (10,000; 100,000; 1,000 000); equivalent aqueous concentrations are shown for solids and PE: *C*_*w,s*_ = *C*_*solids*_/*K*_*d*_ and *C*_*w,PE*_ = *C*_*PE*_/*K*_*PE*_. For desorption from solids, three mass transfer models are used: film diffusion across an external aqueous boundary layer (top), intraparticle pore diffusion (middle), and both mass transfer models coupled (bottom). Uptake in PE is delayed with increasing *K*_*PE*_, but in water, high *K*_*d*_ values lead to faster equilibration (this is specific for finite bath conditions). Parameters: ${D}_{aq}=$ 6.71E-10 m^2^ s^−1^; ${\rho }_{PE}=$ 0.92 kg L^−1^; ${\text{SL}}_{PE}=$ 0.001 kg L^−1^; particle size ${d}_{s}=100$ μm; ${d}_{PE}=80$ µm; ${\delta }_{PE}=$ 170 µm; intraparticle porosity $\varepsilon =$ 0.05; energy dissipation rate ${\varepsilon }_{disp}$ = 10^−1.5^ m^2^ s^−3^; kinematic viscosity of water $\nu$ = 10^−6^ m^2^ s^−1^; Sherwood number: ${Sh}_{solids}$ = 2+ $0.52{Re}^{1/2}{Sc}^{1/3}$  = 9.2 (Armenante and Kirwan, 1989); Reynolds number: $Re={{\varepsilon }_{disp}}^{1/3}{d}_{s}^{4/3}/\nu$; Schmidt number: $Sc=\nu /{D}_{aq}$); equivalent initial aqueous concentration ${C}_{w,\text{s},\text{ini}}=$ 100 $\mu$ g L.^−1^ (for modelling details see Liu et al., 2022)

Passive samplers are also used in laboratory experiments to determine the freely dissolved concentration of pollutants in aqueous suspension containing sediment or soil particles, which is important for the assessment of bioavailability as well as for mobility in water. In such cases, the experimental system involves three phases (solids, water, and passive sampler) with the solid particle phase initially acting as the pollutant source. Here, infinite bath conditions are approached even faster. In order to illustrate this, we may assume suspended particles are in local equilibrium with the water which leads to an extension of the “*V*_*w*_ “ term in Eq. 6 by the dry mass of suspended solids *m*_*d*_ times their respective distribution coefficient (*K*_*d*_):

7 $${t}_{ch}=\frac{{\delta }_{aq}}{{D}_{aq}} \frac{{K}_{PE} {\rho }_{PE} {d}_{PE}}{2 \left(1+{K}_{PE}\frac{{m}_{PE}}{{V}_{w}+{K}_{d} {m}_{d}}\right)}$$

This effect is illustrated in Fig. 5 for different solid to liquid ratios of suspended particles and shows that characteristic times of the infinite bath are approached or even slightly exceeded if desorption kinetics from 100-µm-sized particles are also included (note, *t*_*ch*_ is additive: uptake time in PE + desorption time from the particles); for smaller particles (faster kinetics), the symbols would move closer to the equilibrium lines.

In Fig. 6, we illustrate complex dynamic changes in (equivalent) aqueous concentrations using a theoretical example on pollutant redistribution between three phases (suspended solids, water, passive sampler). Initially, the decrease in concentration in the solids is mirrored by a rapid increase in aqueous concentration leading to an “overshooting” concentration in water not captured by the PE. Uptake in PE is delayed and slows down with increasing *K*_*PE*_ in contrast to the finite bath characteristic times shown in Fig. 5 (which become independent on *K*_*PE*_ if they become large). This becomes more pronounced if the solid to liquid ratio (*m*_*d*_/*V*_*w*_) increases. The suspended solids keep the aqueous concentration initially at a more or less constant high level and thus the PE “experiences” infinite bath boundary conditions causing slower uptake with increasing *K*_*PE*_. This may be also the reason for non-equilibrium conditions observed for high molecular weight PAHs in our partitioning experiments (Fig. 1) because spiking likely initially caused precipitation of solids slowing down uptake in PE (until they got dissolved). As Fig. 6 also shows, kinetics are faster if desorption from the particles is only limited by diffusion through an external aqueous boundary layer; for interparticle diffusion, it is much slower and finally a coupled model has to be considered which accounts for a shift in mass transfer mechanisms (from film diffusion at early times to interparticle diffusion at late times). For more information on this type of “redistribution” modelling, see Liu et al. (2022).

Equations 3–7 and Fig. 5 demonstrate how the equilibration process is influenced by the boundary conditions (e.g., different solid to liquid ratios of PE and suspended solids). This has implications for the sampling rates commonly employed when using passive samplers under non-equilibrium conditions (e.g., Bartkow et al., 2006; Tuduri et al., 2006). Since sampling rates are calculated from the rate constant (or inverse characteristic time, Eq. 6) times the sampler volume and the volumetric partition coefficient (and thus has units in volume/time), they depend on the boundary conditions and are affected by the solid to liquid ratio if measured in batch experiments. This especially affects compounds with high *K*_*PE*_, because rate constants determined in the lab may be independent on *K*_*PE*_ (if larger than *V*_*w*_/*m*_*PE*_). This becomes relevant if kinetic data obtained in the lab are transferred to the field (or different boundary conditions such as solid to liquid ratios) and justifies the use of performance reference compounds which have similar *K*_*PE*_ as the target compounds.

## Conclusions

Our experimental data confirm that uptake and release of Phe on PE is reversible and kinetics are limited by diffusion through the aqueous boundary layer. Thus, a simple mechanistic model allows to determine the thickness of the boundary layer (here 170 µm). Our results also confirm that partitioning of PAHs into PE is independent on concentration and linear over large concentration ranges (three orders of magnitude). However, for high molecular weight PAHs (*K*_*PE*_ > 6), the experimental setup leads to an artifact after initial spiking causing solid precipitates which may have slowed down uptake kinetics onto PE (resulting in underestimation of *K*_*PE*_). For medium and low molecular weight PAHs (*K*_*PE*_ < 6), *K*_*PE*_ values measured correlate well with log *K*_*ow*_, *MW*, and log *S*_*sub*_ and confirm existing empirical relationships from literature.

Uptake and release of pollutants onto and from passive samplers (e.g., PE sheets) may get quite complex if a three-phase system (water, PE, solids) has to be considered. Time scales for equilibration in water typically increase with increasing *K*_*PE*_ in the infinite bath (“field”), while in batch experiments, they become independent on *K*_*PE*_ if it exceeds the water to PE ratio. This is not the case if a third phase is present such as suspended solids (e.g., in soil slurries, sediment suspensions, solid precipitates) which keeps the concentration in water more or less constant (“infinite bath boundary conditions”). Uptake in PE then gets delayed and slows down with increasing *K*_*PE*_; equilibration slows down further with increasing solid to liquid ratio (Fig. 6). Overall, quite unexpected slow kinetics of passive sampling may be observed as demonstrated in Fig. 6 for pollutant redistribution in such three-phase systems depending on the mechanistic model used for solute desorption from solids (film diffusion vs. intraparticle pore diffusion). Notable is the “overshooting” concentration peak in water which is not captured by the PE. Such redistribution phenomena apply commonly for sorption/desorption kinetics of heterogeneous samples in batch systems as already demonstrated by Kleineidam et al. (1999).

## Data Availability

Data is provided within the manuscript.
